# First-pass and high-resolution ECG-gated MRA of the thoracic vasculature in children and adolescents using gadobutrol at 3 T

**DOI:** 10.1186/1532-429X-13-S1-P199

**Published:** 2011-02-02

**Authors:** Darius Dabir, Claas Philip Naehle, Andreas Mueller, Katharina Strach, Hans Schild, Daniel Thomas

**Affiliations:** 1 University of Bonn, Bonn, Germany

## Introduction

Using first-pass MRA (FP-MRA) spatial resolution is limited by breath-hold duration, and image quality (IQ) may be hampered by motion artefacts. Studies at 1.5 T have shown that the use of blood-pool contrast agents (BP-CA) allows for motion-compensated high-resolution MRA during the steady-state, yielding significantly higher IQ compared to FP-MRA. However, MRA with BP-CAs does not allow for viability imaging. At 3 T the use of a slowly injected extracellular CA at double dose may allow for a high-resolution MRA (HR-MRA) AND viability imaging.

## Purpose

The purpose of this study was 1) to implement an ECG-gated free breathhing HR-MRA protocol using an extracellular CA (Gadobutrol) at 3 T and 2) to compare the vessel sharpness and image quality of standard FP-MRA to HR-MRA of the thoracic vasculature in children and adolescents with congenital disorders (CD) or acquired disease (AD) of the thoracic vasculature.

## Methods

To date, 13 Patients (age range: 5 to 41 years) with CD or AD of the thoracic vasculature underwent both, FP-MRA and HR-MRA using an extracellular CA. HR-MRA imaging was part of a comprehensive imaging protocol including viability imaging for assessment of CD and AD. Vessel sharpness of left pulmonary artery (LPA), left superior pulmonary vein (LSPV) and aorta ascendens (AA) were calculated. Furthermore, image quality was rated independently by two readers using a four point grading scale (1: no image artefacts and excellent vessel sharpness; 4) severe artefacts with poor vessel delineation).

## Results

**Figure 1:**
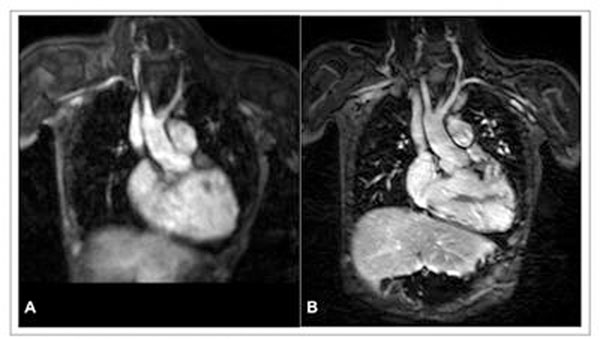
**A:** FF-MRA **B**: HR-MRA

HR-MRA yielded significantly higher vessel sharpness for LSPV (FP-MRA: 0.38 ± 0.11 vs. HR-MRA: 0.57 ± 0.12), LPA (FP-MRA: 0.37 ± 0.06 vs. HR-MRA: 0.50 ± 0.11) and AA (FP-MRA: 0.50 ± 0.10 vs. HR-MRA: 0.74 ± 0.15) compared to the FP-MRA. Image quality was rated higher for HR-MRA compared to FP-MRA by both reviewers. Typical images are displayed in Figure 1, demonstrating the advantage of motion-compensated HR-MRA (B) vs. FP-MRA (A).

## Conclusions

HR-MRA of the thoracic vasculature using extracellular CA offers superior vessel sharpness and improved image quality compared to standard FP-MRA at 3 T. Thus HR-MRA may be used as an add-on to standard FP-MRA. Due to the inherent delayed data acquisition of viability imaging in combination with double dose CA regimen, HR-MRA does not require additional scanning time or contrast agent injection.

